# Systematic medical assessment, referral and treatment for diabetes care in China using lay family health promoters: protocol for the SMARTDiabetes cluster randomised controlled trial

**DOI:** 10.1186/s13012-016-0481-8

**Published:** 2016-08-17

**Authors:** David Peiris, Lei Sun, Anushka Patel, Maoyi Tian, Beverley Essue, Stephen Jan, Puhong Zhang

**Affiliations:** 1 The George Institute for Global Health, Sydney, Australia; 2 The George Institute for Global Health, Beijing, China; 3 The University of Sydney, Sydney, Australia

**Keywords:** Type 2 diabetes, Capacity strengthening, mHealth, China, Implementation

## Abstract

**Background:**

Type 2 diabetes (T2DM) affects 113.9 million people in China, the largest number of any country in the world (JAMA 310:948–59, 2013). T2DM prevalence has risen dramatically from around 1 % in the 1980s to now over 10 % and is expected to continue rising. Despite the growing disease burden, few people with T2DM are achieving adequate management targets to prevent complications. Health system infrastructure in China is struggling to meet these gaps in care, and innovative, cost-effective and affordable solutions are needed. One promising strategy that may be particularly relevant to the Chinese context is improving support for lay family members to care for their relatives with T2DM.

**Methods:**

We hypothesise that an interactive mobile health management system can support lay family health promoters (FHP) and healthcare staff to improve clinical outcomes for family members with T2DM through medical assessment, regular monitoring, lifestyle advice and the prescribing of guidelines recommended medications. This intervention will be implemented as a cluster randomised controlled trial involving 80 communities (40 communities in Beijing and 40 rural villages in Hebei province) and 2000 people with T2DM. Outcome analyses will be conducted blinded to intervention allocation.

The primary outcome is the proportion of patients achieving ≥2 “ABC” goals (HbA1c <7.0 %, blood pressure (BP) <140/80 mmHg and LDL cholesterol <100 mg/dl or 2.6 mmol/L) at the end of follow-up (Diabetes Care 36(Supplement 1):S11-S66, 2013). Secondary outcomes include the proportion of patients achieving individual ABC targets; mean changes in HbA1c, BP, LDL, renal function (serum creatinine and urinary albumin), body mass index, quality of life (QOL, EQ-5D), and healthcare utilisation from baseline; and cost-effectiveness/utility of intervention. Trial outcomes will be accompanied by detailed process and economic evaluations.

****Discussion**:**

The Chinese government has prioritised prevention and treatment of diabetes as 1 of 11 National Basic Public Health Services. Despite great promise for mHealth interventions to improve access to effective health care, there remains uncertainty about how this can be successfully achieved. The findings are likely to inform policy on a scalable strategy to overcome sub-optimal access to effective health care in China.

**Trial registration:**

Clinicaltrials.gov NCT02726100

## Background

### Diabetes disease burden and healthcare quality gaps

China has experienced a dramatic increase in the prevalence of type 2 diabetes (T2DM) from around 1 % in the 1980s to now over 10 %. It is home to the largest number of diabetics in the world [1, 2]. The health system has struggled to keep pace with this rapid rise in disease burden. Despite well-established evidence on effective strategies to reduce death and disability from diabetes, its uptake into routine medical care remains limited [3, 4]. In China, it has been estimated that fewer than 10 % of diabetes patients are achieving target HbA1c, blood pressure and low-density lipoprotein (LDL)-C levels [5]. A study from Chaoyang, the biggest district in Beijing, estimated that only about 2 % of patients with hypertension or diabetes received standard care or management [6]. Exclusive reliance on a highly trained medical workforce to address this is not sustainable. Innovative, scalable implementation strategies are therefore urgently needed.

### Evidence of self-management and interactive technology interventions for T2DM

One promising solution is to increase self-management support and build the capacity of lay family members to provide improved diabetes care. Systematic reviews of self-management strategies have demonstrated improvements in diabetes outcomes with reductions in HbA1c of about 0.5 % [7–9]. There is also growing evidence of the contribution of information technology (IT)-based interventions (internet, mobile, decision support and telemedicine) to improving diabetes self-management. A recent systematic review of 104 studies (60 randomised trials) demonstrated improvements in outcomes in 73 % of studies [10]. Despite these promising outcomes, most self-management and lay-support interventions have generally been restricted to high-income countries. Given the highly varied health system and socio-cultural contexts in China, there are major opportunities to generate evidence of effectiveness from self-management and family-based diabetes interventions that are supported by IT strategies.

### Working with family health promoters

Support for people with chronic diseases in China is traditionally provided by children and grandchildren. Although family members are highly motivated to care for relatives, much of this care is variable in quality and not evidence-informed. In 2008, the family health promoter (FHP) project was commenced, in which a voluntary family member received training in chronic disease management and took responsibility for maintaining the health of the whole family. Community health service practitioners were engaged to deliver professional support to these FHPs [11, 12]. Significant improvements in behaviour change, blood pressure and blood glucose control were achieved. In a study involving 11,192 family members, the FHP intervention was associated with a 29.3 % improvement in knowledge scores at 6 months which was sustained at 12-month follow-up [11, 12]. Two randomised controlled trials (102 patients in Beijing and 220 patients in Zhejiang) showed that FHP-based disease management significantly lowered fasting plasma glucose by 0.7 mmol/l compared to usual care (*p* < 0.01) [13, 14]. Publicly praised by the Beijing government, the FHP initiative was recognised as a “Beijing Government Serving People Project” and has planned to train at least 20,000 FHPs each year since 2011 [15]. Despite the success of the trials, the scale-up phase of the programme is experiencing difficulty in sustainability, primarily due to excessive demands on medical staff to support its uptake. Key problems include: (1) a requirement of staff to attend a 12-class training workshop; (2) competing demands with routine clinical work and (3) provision of training and ongoing support to FHPs once enrolled in the programme.

### Previous research using mobile health

Mobile health (mHealth) technologies offer an unprecedented opportunity to address these three questions. In 2012, there were 380 million smartphone users in China, now surpassing the USA to become the leading country for active Android and iOS subscribers [16]. In a recently completed pilot study in two rural China provinces, we found that around 90 % of patients’ close family members have access to mobile phones, and over 50 % of these are smartphones. The SimCard study tested whether training village doctors in the use of an Android application could help support the management for people with cardiovascular disease (CVD) [17]. The intervention was tested in Tibet and rural India in a recently completed 47 village cluster randomised controlled trial (RCT) and demonstrated a 24 % improvement in prescribing of recommended medications. In India, a trial is currently underway of a multifaceted system involving training community health workers to perform village-based CVD risk screening using an Android tablet and uploading data for review and management by the treating primary healthcare doctor. The intervention is being tested in a stepped-wedge cluster RCT involving 18 primary healthcare centres, 54 villages and over 15,000 people at high CVD risk [18]. In Australia, the TORPEDO trial of a provider-focussed decision support system and quality improvement intervention found a 10 % absolute improvement in CVD risk factor measurement and a 17 % improvement in guideline recommended prescribing of medicines to people at high CVD risk who were not taking these medicines at baseline [19].

In this study, we harness these previous initiatives to offer an innovative strategy for addressing the diabetes epidemic in China—SMARTDiabetes.

### Research methods

SMARTDiabetes will be conducted in two phases, and specific aims are as follows:

- Phase 1: intervention development
  1. To conduct a comprehensive barriers analysis of the existing Family Health Promoter project to understand the opportunities and constraints experienced by patients with T2DM and their families in accessing high quality health care
  2. To develop an evidence-based appraisal of best practice recommendations for the management of T2DM and develop these into a health management algorithm
  3. To take a user-centred design approach with urban and rural patients, family health promoters and medical staff to develop and field-test the health management ‘app’
- Phase 2: implementation and evaluation
  1. To conduct a large-scale cluster randomised controlled trial of the system and determine its clinical impact for people with T2DM
  2. To conduct process and economic evaluations to understand intervention impact on patients, FHPs and staff and to determine cost-effectiveness and scale-up opportunities

## Methods/design

### Methods—phase 1

#### Barrier analysis

A comprehensive barriers analysis of the existing Beijing FHP project will be conducted and expanded to one rural province (Hebei) to understand contextual constraints on provision of best practice care. Normalisation Process Theory [20] and the Behaviour Change Wheel [21] will be used to understand the extent to which the intervention was incorporated into routine practice and to assess the capabilities, opportunities and motivation of medical staff, families and patients to obtain improved outcomes related to diabetes. Data sources will include document analysis, process mapping work with stakeholder groups and semi-structured individual interviews.

#### Incorporation of clinical guidelines

Best practice recommendations will be provided based on Chinese national and international guidelines to support self-monitoring of blood pressure and blood glucose, lifestyle change (smoking cessation, weight loss, improved diet, aerobic exercise, alcohol and sodium restriction), adherence to medical treatments and prevention of complications. These will then be programmed into a prototype application for use via desktop computer and mobile device and validated using methods that we have used previously [22].

#### User-centred prototype development

We will then engage platform users (FHPs, patients, doctors and nurses) in the design and development of the prototype applications. User scenarios will be clarified, and the routine workflow patterns that will best engage medical staff and FHPs will also be determined. An agile software development approach will be taken in which prototypes are developed, tested and rapidly re-deployed based on user feedback. Over several cycles, the prototype will progress from low-fidelity (paper-based) concepts to an eventual high-fidelity clickable prototype that will resemble the final product. Once the prototype is finalised, software development of the complete solution will be conducted.

#### EHR integration

In Beijing, a large provider of community health service electronic platforms will be involved in the development of SMARTDiabetes. In Hebei province, we propose a hybrid approach utilising a stand-alone cloud-based system where electronic health record (EHR) uptake is low and an integrated system where EHRs are readily used.

#### Feasibility study and implementation optimisation

**Fig. 1:**
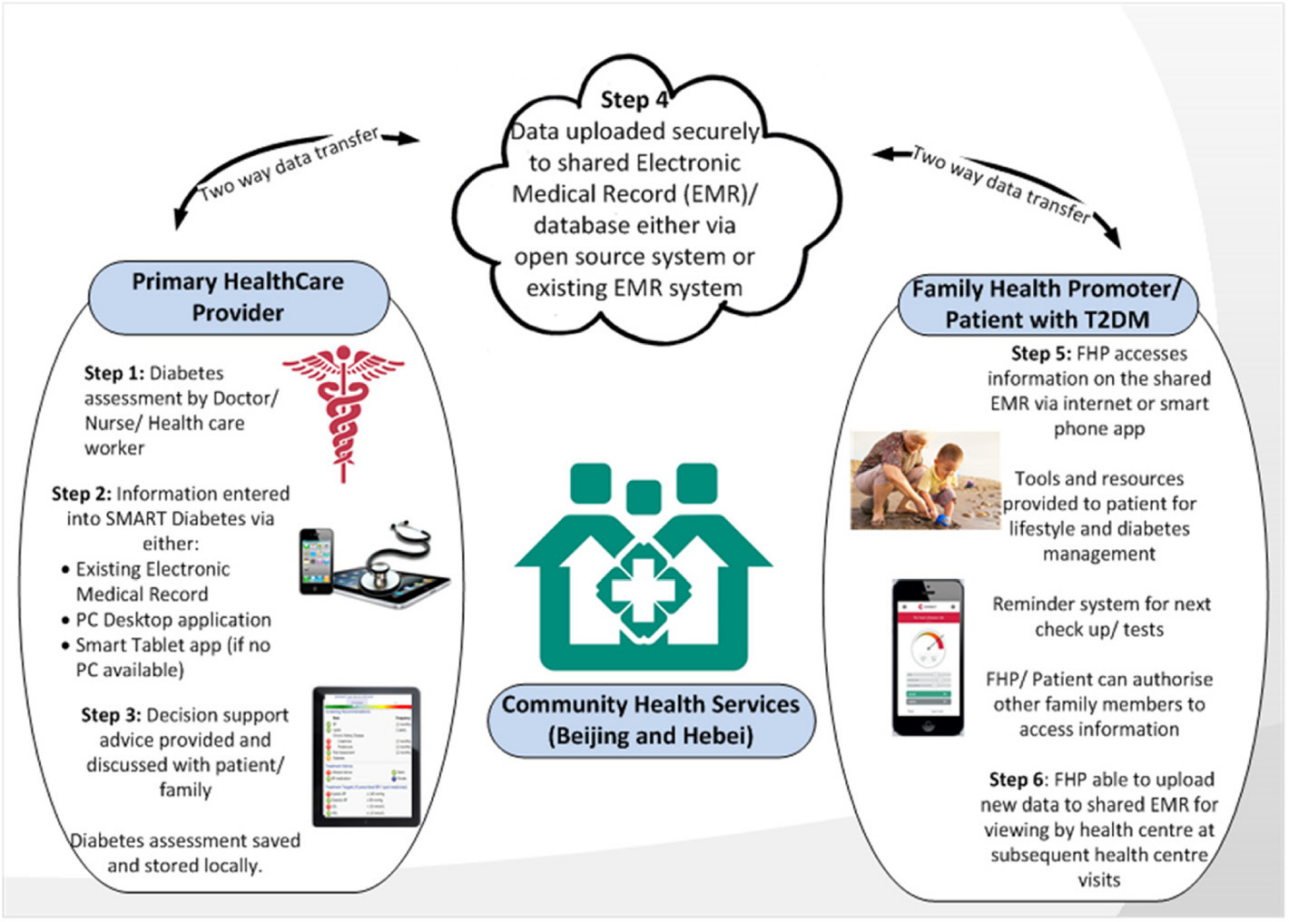
Key elements of the intervention package

Two communities will be selected from Beijing and Hebei to pilot test its feasibility, validity and stability of the platform. Although the exact usage scenarios will not be clarified until the above scoping work is completed, a typical work flow pattern might include the following features (Fig. 1):

- A training and support package will be developed to guide healthcare workers and FHPs on use of the technology and management of diabetes.
- An initial medical consultation with a nurse or doctor will be scheduled, and personalised health information will be entered into either a stand-alone application or directly into the local EMR used at the health service. An assessment of micro- and macrovascular risk, hypoglycaemic risk and management principles will then be generated. The assessment will then be uploaded to a cloud-based electronic medical record.
- The FHP will download the consumer version of the SMARTDiabetes application and register as a user. The application will be available on a mobile device or as a web page on a desktop computer. The FHP will then create an account for the family member whom they would like to look after.
- The consumer version of the SMARTDiabetes app will pre-populate with information from the cloud-based EMR. An action plan wizard will assist FHPs in making personalised health goals and targets for their relatives. Automatic prompts and warnings will be triggered by SMARTDiabetes based on clinical needs, the co-determined action plan and the patients’ progress. Adjustments of action plan could be made any time by the FHPs on behalf of the patient, and data in the EHR will be refreshed with the latest information.
- A forum feature will also be built where multiple users within the community will be able to share experiences with one another.
- The patient’s nominated care provider will also be given an authorised view of the application to assist with health care for use at subsequent health centre visits. All routine medical care will otherwise continue as usual.

Following field testing, the intervention will be optimised in preparation for trial implementation.

### Methods—phase 2

#### Hypothesis

An interactive mobile health management system can support lay family health promoters and healthcare staff to improve clinical outcomes for family members with T2DM, and this system will be affordable, acceptable and potentially scalable across China.

#### Design

**Fig. 2:**
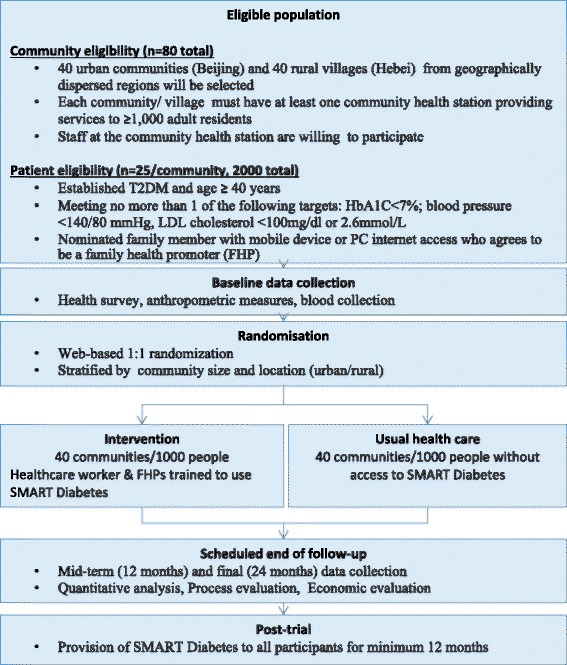
Study schema

This study is a community-based parallel-arm cluster RCT (Fig. 2).

#### Setting

The trial will involve 2000 participants from 80 communities in one urban (Beijing province) and one rural setting (Hebei province). Geographically dispersed communities will be selected within provinces to minimise contamination risk. Each community health station must serve at least 1000 adult residents, and clinical staff at the station must be willing to participate in the trial.

#### Participants eligibility

Eligible participants will then be recruited from these sites. They must have established T2DM, be aged 40 years and above and be meeting no more than one of the following American Diabetes Association “ABC” management targets (HbA1C < 7 %; blood pressure < 140/80 mmHg, LDL cholesterol < 100 mg/dl or 2.6 mmol/L). Participants must also be able to provide informed consent and have a nominated family member who is willing to serve as FHP and has access to a smartphone or internet-enabled desktop computer. The FHP need not be living with the family member. Given many parents, particularly in rural villages, have children working in cities, this criterion will enhance recruitment feasibility and external validity.

#### Randomisation

Communities will be centrally randomised in a 1:1 allocation, stratified by community size and location (urban/rural) to either receive the intervention or continue with usual health care.

#### Intervention

Participants in intervention communities will receive the SMARTDiabetes intervention as defined in Fig. 2 plus usual health care for 24 months. Medical staff and FHPs in the intervention communities will be provided with an initial training session on the installation and use of the platform. These staff will also register as healthcare providers in the system and will be able to track patient progress and provide support to FHPs via communication tools built into the application.

#### Control arm

Patients in the control arm communities will receive usual care provided by community health service system or hospitals. The only exception is that they will also be invited to take part in the survey and examination at baseline, mid-term (12 months) and end of study (24 months) for the purpose of evaluation. To avoid contamination of intervention, the SMARTDiabetes application will only be accessible for FHPs and medical staff in intervention communities via a secure password-protected registration process.

#### Data collection

We will invite participants in both the intervention and control groups to visit the nearby Community Health Service Center to receive a comprehensive survey, standard anthropometric measures and blood collection at baseline, mid-term (12 months) and end of study (24 months). In both Beijing and Hebei, a qualified central laboratory will be selected to conduct standard laboratory tests for all the participants. Table 1 below outlines the data that will be collected. Although it will be difficult to ensure complete blinding of data collectors to community allocation status, these staff will not be involved in the implementation of the intervention. Primary outcome data will be obtained from objective reports (e.g. printable blood pressure readings and pathology laboratory readings), and a proportion of the total sample will be audited for accuracy.

**Table 1:** Data collection procedures

Assessment description	Screening	Baseline/randomisation	12-month visit	Final visit
Informed consent	X			
Eligibility	X	X		
Reasons for non-participation	X	X		
Demographics, medical history,		X		
Medications, physical examination, vital signs, vital status^a^		X	X	X
Quality of life questionnaire		X	X	X
Laboratory results^a^		X	X	X
Serious adverse events		X	X	

^a^End point evaluation data derived from these data elements

#### Primary outcome

The primary outcome is the proportion of patients achieving at least two “ABC” goals (HbA1c <7.0 %, blood pressure (BP) <140/80 mmHg and LDL cholesterol <100 mg/dl or 2.6 mmol/L) [23] at the end of follow-up.

#### Secondary outcomes

Secondary outcomes include the proportion of patients achieving individual ABC targets; mean changes in HbA1c, BP, LDL, renal function (serum creatinine and urinary albumin), body mass index (BMI), quality of life (QOL, EQ-5D), and healthcare utilisation from baseline; and cost-effectiveness/utility of intervention (see economic evaluation).

#### Statistical considerations

We have assumed a baseline T2DM prevalence of 12 % [2] based on published studies [5] and the 2011 Beijing NCD risk factor surveillance data (unpublished data). Assuming 20 % of people in the control arm will achieve ≥2 ‘ABC’ diabetes goals (primary outcome) at the end of the study, a conservative intra-class correlation coefficient of 0.05 (ICC 0.01 in Beijing surveillance data), a 20 % loss to follow-up and a two-sided significance level of 0.05, 80 community clusters and a mean community cluster size of 25 participants (2000 total) will provide 90 % power to detect an absolute improvement of 10 % in the primary outcome in the intervention arm (i.e. 30 vs 20 %). This translates to a mean reduction of 0.35 % for HbA1C, 0.14 mmol/L for LDL cholesterol and 3.4 mmHg for systolic BP. Improvements of this magnitude are clinically relevant and informed by the effect sizes for glycaemic control seen in the previous FHP trial [13] (0.7 mmol blood glucose reduction or ~0.5 % HbA1c reduction). Primary analyses will be conducted at the patient level. Secondary analyses will be conducted at the cluster level. Sub-group analyses will be conducted at the community level (based on size and health service characteristics) and patient level (based on demographic factors (co-habitation with FHP) and clinical factors (control rate of ‘ABC’ risk factors at baseline).

#### Process evaluation

In the process evaluation, the theories used to inform the barriers analysis in phase 1 will be utilised again. Normalisation process theory (NPT) [20] and behaviour change theory [21] will be used to provide an overarching framework to help guide and structure our approach to understanding intervention impact. This will help identify factors that promote and inhibit the incorporation of a complex intervention into routine practice. This is a critical aspect of the project as it will build on the findings of the previous FHP research in determining the scalability of the model. NPT identifies four main components: (1) coherence (sense-making), (2) cognitive participation (engagement), (3) collective action (work done to make the intervention happen) and (4) reflexive monitoring (appraisal of the benefits of the intervention). The broad questions which we will explore under each of these components are outlined in Table 2.

**Table 2:** Normalisation Process Theory constructs [20]

Coherence (i.e. sense making by participants)	Cognitive participation (i.e. commitment and engagement by participants)
• Is the intervention easy to describe? • Is it clearly distinct from other interventions? • Does it have a clear purpose for participants? • Is there a shared sense of its purpose? • What are the benefits and for whom? • Are these benefits likely to be seen as valuable? • Will it fit with the overall goals and activity of the community health service?	• Are target user groups likely to think it is a good idea? • Will they see the point of the intervention easily? • Will they be prepared to invest time, energy and work on it?
Collective action (i.e. the work participants do to make the intervention function)	Reflexive monitoring (i.e. participants reflect on or appraise the intervention)
• How did the intervention affect the work of user groups? • How compatible was it with existing practices? • What effect did it have on clinical care? • Did staff/FHPs/patients require extensive training in order to use it? • What impact did it have on division of labour, resources, power, and responsibility between different professional and community groups? • Did it fit with the overall goals and activity of the community health service?	• How did users perceive the intervention once it had been in use for a while? • Was it likely to be perceived as advantageous for patients or staff? • Was it clear what effects the intervention had? • Were users/staff able to contribute feedback about the intervention once it was in use? • How adaptable was the intervention on the basis of user experience and feedback?

We will take a case study approach in which a purposive sample of community clusters will be selected to maximise variation in characteristics such as size, urban/rural, health service characteristics and baseline performance for the primary outcome. Based on prior experience, we expect that around 12 case communities will be established from the total sample of 80 communities. Several data sources will be obtained in order to inform the impact of the intervention at each of these case study sites and will comprise site-specific analyses of trial data, usage analytics of the website and mobile applications, quantitative satisfaction and feasibility surveys using validated instruments such as the standardised system usability scale [24] and in-depth, semi-structured interviews with health service staff, FHPs and patients. Survey and interview instruments will be iteratively developed, guided by our overarching theories and tested prior to implementation. A mixed methods analysis of quantitative and qualitative data will be conducted by a multidisciplinary team comprising the lead academics and service managers.

#### Economic evaluation

The economic evaluation will have a trial-based component and a modelled evaluation of long-term costs and outcomes. Intervention costs will be based on salaries, training and software development. As we do not expect an effect of the intervention on survival, the (trial-based) incremental cost-effectiveness ratio will be determined by average differences in utility (from the EQ5D questionnaire) observed between treatment arms in the trial. To capture costs and outcomes beyond the trial, a decision-analytic model will be developed to enable long-term morbidity, quality of life and survival to be simulated. The model will specify a number of health states (such as myocardial infarction, stroke, renal failure, type 2 diabetes with and without micro and macro-vascular complications, death and no disease) between which individuals cycle each year over a lifetime. It will draw on the literature and available databases to determine the transition probabilities between health states and the cost and quality of life associated with each. All patients will simulate progression across states until death. Incremental cost per quality-adjusted life year gained will be determined by then folding back the model to determine the average costs and outcomes, discounted at appropriate rates, accrued in both treatment arms. Sensitivity analysis will be conducted to determine the robustness of base case estimates to assumptions used in the economic evaluation. This will better inform policy makers as to the resource consequences of rolling out this programme to scale.

### Trial status

Phase 1 of the study will commence mid-2016. Phase 2 recruitment will commence recruitment mid-2017.

## Discussion

The Chinese government has placed prevention and treatment of diabetes as one of 12 National Basic Public Health Services [25]. In 2015, around 50 billion yuan (US $8.2 billion) was allocated to support these services and policy makers are seeking solutions to address diabetes-related disease burden [25]. Despite great promise for mHealth interventions to improve access to effective health care, there remains uncertainty about how this can be successfully achieved. These uncertainties pose substantial dilemmas for health system planners, particularly in limited resource settings. Our proposal will comprehensively explore the challenges of implementing well-established evidence into practice.

### Strengths and limitations

The strengths of the study are that it explores the challenges of implementing a complex intervention, taking into consideration policy, healthcare providers and consumer perspectives.

The main limitation is that it is conducted in one rural and one urban region of China, and the findings may not be immediately generalizable to other health system contexts particularly where electronic health record infrastructure varies and access to smartphone technology may be limited. The focus of the study on integrating the intervention within the existing primary healthcare system, which is broadly similar across China, will help to mitigate this and enhance the relevance to other regions with similar health system structures.

### Significance

The proposed project involves key decision-making partners from the Management Center for Community Health Services in two provinces which will be critical for potential scale-up considerations. Although focusing on T2DM, the platform developed and the model of care proposed could support the management of multiple chronic diseases. Consequently, we expect the findings to advance locally relevant knowledge on strategies to support the health system in both urban and rural areas of China. This research is at the intersection of policy, industry, health care providers and consumers and seeks to ensure that the evidence generated has the maximum potential to inform decision-making for system planners. It has the potential to close significant gaps in treatment experienced by many millions of individuals with diabetes in China.

## Abbreviations

BP, blood pressure; EHR, electronic health record; EQ5D, European Quality of Life 5-Item questionnaire; FHP, family health promoter; HbA1C, glycated haemoglobin; LDL, low-density lipoprotein; QOL, quality of life; RCT, randomised controlled trial; T2DM, type 2 diabetes
